# Different Types of Resistant Starch Elicit Different Glucose Reponses in Humans

**DOI:** 10.1155/2010/230501

**Published:** 2010-01-05

**Authors:** Mark D. Haub, Kelcie L. Hubach, Enas K. Al-tamimi, Sammy Ornelas, Paul A. Seib

**Affiliations:** ^1^Human Metabolism Laboratory, Department of Human Nutrition, Kansas State University, Manhattan, KS 66506, USA; ^2^Department of Grain Science and Industry, Kansas State University, Manhattan, KS 66506, USA

## Abstract

The purpose of this study was to determine whether different types of resistant starch (RS) elicited different glycemic responses. Eleven healthy subjects consumed solutions containing 30 g of either dextrose (DEX), resistant starch type 2 (RS2), or cross-linked resistant wheat starch type 4 (RS4_XL_) on three separate occasions, which were assigned randomly. Finger stick blood samples were collected before and over the following two hours and measured for glucose. The incremental area under the curve (iAUC) for the glucose response was calculated for all trials. The two types of resistant starch significantly (*P* < .05) decreased iAUC compared with DEX. The response with RS4_XL_ was significantly decreased compared with the RS2 trial. These data demonstrate that different types of resistant starch elicit significantly different glycemic responses.

## 1. Findings

Foods containing resistant starch (RS) generally give a low glycemic response because RS is not digested in the small intestine. Instead RS passes into the large intestine where it is fermented [1–8]. Starch can escape digestion if it is embedded in a matrix that renders the starch inaccessible to enzymes (type 1, RS1). In addition, some untreated starch granules are known to resist digestion (RS2). Again, starch which is cooked and cooled, nongranular, and sometimes debranched, forms RS when it reassociates and recrystallizes (RS3). Finally, starches that are structurally modified become resistant (RS4). 

Examples of RS2 also include untreated granules of potato, green bananas, and high-amylose maize starches. When boiled in water, potato, and banana starches lose their resistance, but high-amylose maize starch, being difficult to cook, partially retains granular structure and resistance to digestion. Hydrothermal treatment of high-amylose maize starch enhances its level of RS [7, 9]. Examples of RS4 are cross-linked starches [10–12], starch esters [13], starch ethers, [14] and pyrodextrins with new glycosidic linkages differing from alpha-1, 4 and alpha-1, 6 bonds [7, 15]. 

The swelling and solubilities of RS2 from high-amylose (70%) maize starch and RS4_XL_ are much lower than normal cereal starches in both cold and hot water [11, 16, 17]. At 95°C in excess water RS4_XL_ showed a swelling power of 2.8 g/g and solubility of 0.5% compared to untreated wheat starch at 7.6 g/g and 27.7% [17]. Hydrothermally treated high-amylose (70%) maize starch (Novelose 240, which contains less RS than Novelose 260) did not change granular structure when heated in water up to 85°C [16], and it showed a swelling power at 95°C of 2.1 g/g and solubility of 1.9% [17]. 

Most clinical trials to date have used RS2 as the ingredient of choice for studies investigating the glucose lowering potential of RS. There is a paucity of research on the clinical outcomes of other types, especially RS4. Wheat-derived RS4_XL_ may have greater potential at decreasing the glucose response as it contains a high degree of dietary fiber and RS [18], but there has only been one clinical trial testing its efficacy at lowering blood glucose [19]. Furthermore, there are limited, if any, studies that have compared the health outcomes of various types of RS, which makes it difficult to fully understand the beneficial capacity of RS to assist with glucose control. Thus, the aim of this clinical trial (NCT00930956; ClinicalTrials.gov) was to determine whether RS2 (the most commonly tested type of RS) and RS4_XL_ elicit similar glucose responses.

## 2. Research Design and Methods

The volunteers (females *n* = 7, males *n* = 4; age = 24 ± 4 yr; ht = 1.65 ± 0.07 m; wt = 63.7 ± 13.1 kg; BMI = 23.2 ± 3.8 kg/m^2^) were not diagnosed with any chronic disease. The Institutional Review Board of Kansas State University approved the study, and all volunteers provided written informed consent. 

Each volunteer visited the laboratory in a 10–12 hour fasted state on three occasions over a three-week period, up to two visits/wk, with at least 48 hours between visits. Volunteers were asked to refrain from vigorous physical activity and the consumption of alcohol the day before each testing visit. Randomization using a Latin Square design was applied to minimize confounding issues associated with the order of administration. Each volunteer consumed 30 g of carbohydrate in the following forms: 178 mL of a dextrose beverage (DEX; Sun-Dex); resistant starch type 2 diluted in 178 mL of water (RS2; Hi-Maize 260); cross-linked resistant wheat starch diluted in 178 mL of water (RS4_XL_; Fibersym RW). The dose was established from a prior study showing improved insulin sensitivity using RS2 [20]. The use of the DEX treatment was to provide a reference from which to compare the other treatments, and allow for determining a relative glycemic response. 

In the morning of each test, finger-prick capillary blood samples were collected to determine fasting (baseline) blood glucose levels. The volunteers then consumed the test solution assigned for that trial. Ten minutes were allowed for the test solution to be consumed. Over the two hours following the start of each test, finger-prick capillary blood samples were collected at 30, 60, 90, and 120 minutes. Blood glucose levels were immediately measured in duplicate using an automated blood glucose analyzer (YSI 2300, Yellow Springs, Ohio, USA). Analysis of the collected sample was repeated if the difference between duplicate samples was greater than 0.1 mmol/L. Once the samples were analyzed, the data was entered and the incremental area under the curve was calculated using the trapezoidal model (GraphPad v5.0, La Jolla, Calif, USA).

**Statistical Analysis**

Paired *t*-tests were used to determine significant differences between treatments at each time point and to determine differences between iAUC values; significance was set at *P* = .05. The data was statistically analyzed using SPSS software (v13.0; Chicago, Ill, USA).

## 3. Results

**Figure 1:**
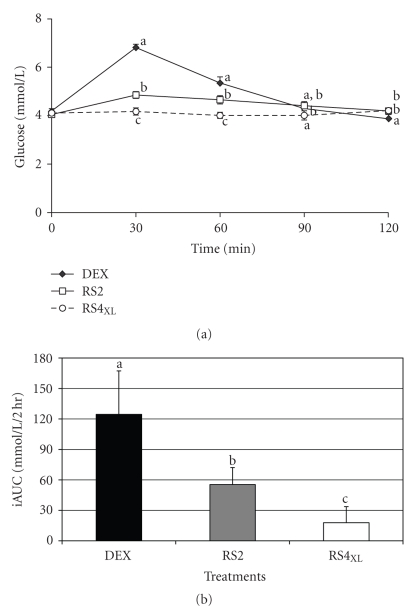
The glucose responses to 30 g of carbohydrate from three treatments (DEX, RS2 and RS4_XL_). Panel (a) depicts the glucose changes over time, while panel (b) depicts the incremental area under the glucose curve. Data presented are mean±SE; significance was set at *P* < .05; and, different letters indicate difference between treatments.

The numerical peak in glucose for the DEX and RS2 treatments occurred at 30 minutes, while the glucose peak during the RS4_XL_ treatment did not occur until 120 minutes (Figure 1(a)). The iAUC for each treatment was different from one another (Figure 1(b)). The DEX trial elicited an increased iAUC compared with RS2 (*P* = .001) and RS4 (*P* = .000), while the iAUC for RS2 was increased compared with RS4_XL_ (*P* = .002). The relative glycemic responses were 100%, 34.9 ± 11%, and 11.3 ± 10% for DEX, RS2, and RS4_XL_, respectively.

## 4. Conclusions

This randomized clinical study demonstrates that different types of RS elicit different glycemic responses. This study supports prior studies where RS attenuated the glucose response [19–25], as both types of RS in the present study attenuated the glucose response compared with the same amount dextrose. However, these data indicate that not all RS types elicit similar glucose responses, as the RS4_XL_ response was less than RS2. A reason for this observation is likely that RS4_XL_ contains a greater degree of dietary fiber (91.9%) and more RS (83%) [18] compared with the fiber (60%) and RS content (46%) of the version of RS2 that was used [26]. This is a critical application issue since companies use these functional starches as ingredients in food products. As an ingredient, these results indicate that based on a weight comparison, the RS4_XL_ elicits a greater capacity to attenuate the glucose response. Additionally, it has previously been demonstrated that RS4_XL_ attenuates blood glucose and insulin responses when incorporated in food [19]. 

This study is limited by the fact that the volunteers consumed the ingredients as is which this is not the form that these ingredients are typically consumed. However, to determine glucoregulatory differences between types of RS, this approach of direct unprocessed comparison is necessary to determine how processing or cooking can affect the level of RS remaining in a food [18]. Future studies should compare other types of raw RS to determine which offers the greatest glucose lowering potential. Additionally, these different types need to be compared in typical food applications. All in all, both types of RS elicited a lower blood glucose response in vivo; however, the type of RS needs to be considered because the glucose lowering capacity can differ significantly.

## Abbreviations

DEX: Dextrose trial
iAUC: Incremental area under the curve
RS: Resistant starch
RS2: Resistant starch type 2
RS4: Resistant starch type 4
RS4_XL_: Cross-linked resistant wheat starch, type 4 RS.

## References

[1] Brown IL, McNaught KJ, Andrews D, Morita T, McCleary BV, Prosky L (2001). Resistant Starch: plant breeding, applications, development and commercial uses. *Advanced Dietary Fibre Technology*.

[2] Englyst KN, Liu S, Englyst HN (2007). Nutritional characterization and measurement of dietary carbohydrates. *European Journal of Clinical Nutrition*.

[3] Higgins JA, Higbee DR, Donahoo WT, Brown IL, Bell ML, Bessesen DH (2004). Resistant starch consumption promotes lipid oxidation. *Nutrition and Metabolism*.

[4] Jenkins DJA, Kendall CWC (2000). Resistant starches. *Current Opinion in Gastroenterology*.

[5] Nugent AP (2005). Health properties of resistant starch. *Nutrition Bulletin*.

[6] Sajilata MG, Singhal RS, Kulkarni PR (2006). Resistant starch—a review. *Comprehensive Reviews in Food Science and Food Safety*.

[7] Thompson DB, Biliaderis CG, Izydorczyk M (2007). Resistant starch. *Functional Food Carbohydrates*.

[8] Sharma A, Yadav BS (2008). Resistant starch: physiological roles and food applications. *Food Reviews International*.

[9] Shi YC, Jeffcoat R, McCleary BV, Prosky L (2008). Structural features of resistant starch. *Advanced Dietary Fibre Technology*.

[10] Aparicio-Saguilan A, Gutierrez-Meraz F, Garcia-Suarez FJ, Tovar J, Bello-Perez LA (2008). Physicochemical and functional properties of cross-linked banana resistant starch. Effect of pressure cooking. *Starch*.

[11] Woo KS, Seib PA (2002). Cross-linked resistant starch: preparation and properties. *Cereal Chemistry*.

[12] Xie X, Liu Q (2004). Development and physicochemical characterization of new resistant citrate starch from different corn starches. *Starch*.

[13] Clarke JM, Bird AR, Topping DL, Cobiac L (2007). Excretion of starch and esterified short-chain fatty acids by ileostomy subjects after the ingestion of acylated starches. *American Journal of Clinical Nutrition*.

[14] Azemi BMNM, Wootton M (1984). Invitro digestibility of hydroxypropyl maize, waxy maize and high amylose maize starches. *Starke*.

[15] Ohkuma K, Wakabayashi S, McCleary BV, Prosky L (2001). Fibersol-2: a soluble, non-digestible, starch-derived dietary fiber. *Advanced Dietary Fibre Technology*.

[16] Ratnayake WS, Jackson DS (2008). Thermal behavior of resistant starches RS 2, RS 3, and RS 4. *Journal of Food Science*.

[17] Shin M, Woo K, Seib PA (2003). Hot-water solubilities and water sorptions of resistant starches at 25°C. *Cereal Chemistry*.

[18] Yeo LL, Seib PA (2009). White pan bread and sugar-snap cookies containing wheat starch phosphate, a cross-linked resistant starch. *Cereal Chemistry*.

[19] Al-tamimi EK, Seib PA, Snyder BS, Haub MD (2010). Consumption of cross-linked resistant starch (RS4_XL_) on glucose and insulin responses in humans. *Journal of Nutrition and Metabolism*.

[20] Robertson MD, Bickerton AS, Dennis AL, Vidal H, Frayn KN (2005). Insulin-sensitizing effects of dietary resistant starch and effects on skeletal muscle and adipose tissue metabolism. *American Journal of Clinical Nutrition*.

[21] Heacock PM, Hertzler SR, Wolf B (2004). The glycemic, insulinemic, and breath hydrogen responses in humans to a food starch esterified by 1-octenyl succinic anhydride. *Nutrition Research*.

[22] Nilsson AC, Ostman EM, Holst JJ, Bjorck IME (2008). Including indigestible carbohydrates in the evening meal of healthy subjects improves glucose tolerance, lowers inflammatory markers, and increases satiety after a subsequent standardized breakfast. *Journal of Nutrition*.

[23] Reader DM, O’Connell BS, Johnson ML, Franz M (2002). Glycemic and insulinemic response of subjects with type 2 diabetes after consumption of three energy bars. *Journal of the American Dietetic Association*.

[24] Robertson MD, Currie JM, Morgan LM, Jewell DP, Frayn KN (2003). Prior short-term consumption of resistant starch enhances postprandial insulin sensitivity in healthy subjects. *Diabetologia*.

[25] Yamada Y, Hosoya S, Nishimura S (2005). Effect of bread containing resistant starch on postprandial blood glucose levels in humans. *Bioscience, Biotechnology and Biochemistry*.

[26] Le Leu RK, Hu Y, Brown IL, Young GP (2009). Effect of high amylose maize starches on colonic fermentation and apoptotic response to DNA-damage in the colon of rats. *Nutrition and Metabolism*.

